# End-of-life care in the emergency department for the patient imminently dying of a highly transmissible acute respiratory infection (such as COVID-19)

**DOI:** 10.1017/cem.2020.352

**Published:** 2020-03-26

**Authors:** Ariel Hendin, Christian G. La Rivière, David M. Williscroft, Erin O'Connor, Jennifer Hughes, Lisa M. Fischer

**Affiliations:** * University of Ottawa Department of Emergency Medicine, Ottawa, ON; ‡ University of Ottawa Department of Medicine, Division of Critical Care, Ottawa, ON; † University of Manitoba Department of Emergency Medicine and Department of Family Medicine, Section of Palliative Care, Winnipeg, MB; § University of British Columbia Department of Emergency Medicine and Division of Palliative Care, Vancouver, BC; ¶ University of Toronto Department of Medicine, Divisions of Emergency Medicine and Palliative Medicine, Toronto, ON; # University of Calgary Department of Emergency Medicine and Division of Palliative Medicine, Calgary, AB; ‖ University of Ottawa Department of Medicine, Division of Palliative Care, Ottawa, ON

**Keywords:** COVID-19, end of life, palliative care, pandemic

Coronavirus-19 disease (COVID-19) has quickly spread to cause a global pandemic, and produces a spectrum of disease from mild respiratory illness to severe acute respiratory distress syndrome. Current estimates indicate that 15% of patients with COVID-19 will develop severe disease, and 5 to 10% will require intensive care-level support. In certain scenarios, escalation of life-sustaining therapies (defined as intubation, mechanical ventilation, vasopressor support, and/or hemodialysis) will either not be within the patient's goals of care, or will unfortunately be unsuccessful. Overall mortality risk from COVID-19 is estimated to be between 3 and 5%.^1,2^

Decision-making around goals of care should, as always, be patient-centered and addressed early in the patient's illness trajectory. Concerns around overall resource use in COVID-19 should not affect individualized decision-making in the absence of clear guidance from administrators and ethicists. As the pandemic evolves, decisions around distributive justice and resource use may become necessary; however, this document focuses on the care of the individual patient before the emergency physician (EP).

Here, we provide a framework for health care providers caring for emergency department (ED) patients with confirmed or suspected COVID-19 who are nearing end of life. The safety and health of care providers and family members of a patient with COVID-19 must be carefully balanced with meticulous symptom assessment and management to allow the patient to die comfortably and with dignity.

Care of the imminently dying patient should not differ significantly from standard best palliative care practices, but there are some pertinent modifications in COVID-19 to consider with respect to:

○ Nonpharmacological management
○ Pharmacological management
○ Withdrawal of life sustaining treatments
○ Support for staff who are providing end-of-life care

The recommendations in this document were based on best evidence where available and by consensus from Canadian physicians who practice both Emergency and Palliative Medicine.

## For all patients

• Document the discussion around goals of care with the patient and/or substitute decision makers and update the patient's category status in the medical record.
• Consider involving Spiritual Care, Social Work, and/or Palliative Care if appropriate. Ensure you have communicated the patient's COVID-19 testing status, whether confirmed or pending, so that all providers are aware of the need for appropriate personal protective equipment.
• Place the patient in a private room if possible, with clear instructions to follow contact and droplet precautions.
• Visitation
  ○ Due to COVID-19, visitation in most centers is being restricted. Please refer to the most up-to-date local protocols.
  ○ When visiting, family members must follow droplet and contact precautions, including wearing a procedure mask with face shield, isolation gown, and gloves, and perform hand hygiene before and after their visit.^3^
  ○ Encourage visits with relatives by means of telephone or video conferencing, if possible, to minimize physical visitors.

## Nonpharmacological symptom management

• Recognize that nursing assessments of patients dying of highly transmissible acute respiratory infections are intensive, time consuming, and require a high degree of cognitive load. This will likely require a lower patient to nurse ratio and/or frequent relief of nursing duties.
  ○ Assessments will involve:
    ■ Frequent symptom assessments using validated tools for signs of distress (pain, agitation, dyspnea) and provision of medication as appropriate for symptoms.^4–6^
    ■ Frequent patient repositioning.
    ■ Eye and mouth care **(avoiding deep suctioning)**.
    ■ Emotional support to patient and family.
• Review all medications and discontinue those not contributing to patient comfort.
• Discontinue devices not necessary for comfort or medication administration (i.e., monitors, nasogastric tubes, additional intravenous lines).
• Discontinue or minimize intravenous fluids and enteral feeding as this does not contribute to patient comfort nearing end of life.^7,8^ If the decision is made to continue enteral feeding of intravenous fluids, monitor closely for complications, including aspiration and pulmonary or peripheral edema.
• Consider insertion of a subcutaneous lock for medication delivery.
• **Avoid the use of the following as they may generate aerosolized SARS-CoV2 virus particles and infect health care workers and family members.**^9–12^
  ○ Fan
  ○ Oxygen flow greater than 6 L/minute
  ○ High-flow nasal cannula oxygen
  ○ Continuous positive airway pressure (CPAP) or bilevel positive airway pressure (BiPAP)
  ○ All nebulized treatments (bronchodilators, epinephrine, saline solutions, etc)

## Pharmacologic symptom management

While the information in online Appendix 1 provides recommended dosing of medications to manage common symptoms patients experience at end of life, dosing and frequency of medication administration should be individualized based on the patient's response. Consultation with Palliative Care is recommended if there is difficulty in managing symptoms.

## Withdrawal of life-sustaining therapy

**Figure 1.**
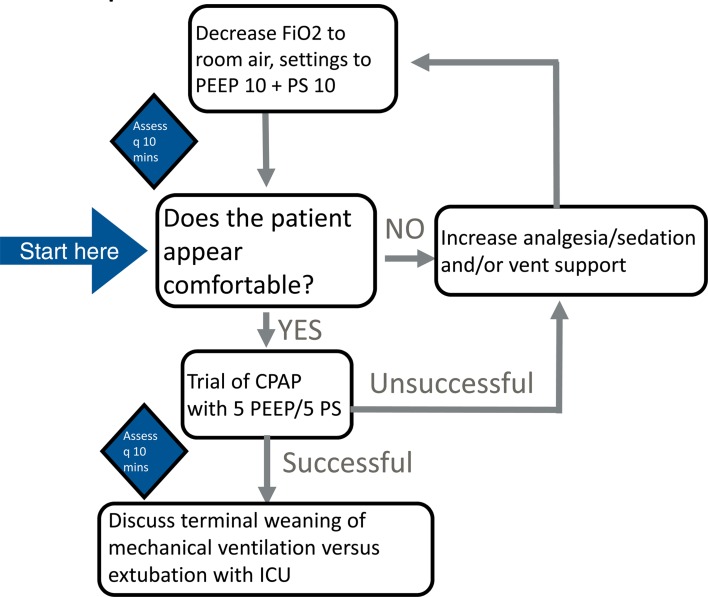
Approach to withdrawal of mechanical ventilation in the patient with suspected or confirmed COVID-19 in the ED.

There will be instances where decisions will be made to withdraw life-sustaining therapy, such as mechanical ventilation. Figure 1 provides a simplified overview of initial steps in weaning mechanical ventilatory support while ensuring patient comfort, but please also refer to local institutional resources where available for best practices to wean ventilatory support.

Given that extubation is considered an aerosol generating procedure and thus can be high risk to health care workers and family members present in the room, our recommendation is to **not extubate the patient in the ED, but to decrease ventilatory support and ensure comfort throughout** (see Appendix). If extubation is being considered, the patient should be in a negative pressure room, and all providers should be prepared with airborne personal protective equipment. Before this, we recommend speaking with experts in Critical Care and following best practices for withdrawal of mechanical ventilation.

## Support to staff who are providing end-of-life care

Being responsible for decisions around resource rationing and use, on top of witnessing an increased frequency of suffering and death means ED health care workers are at heightened risk of burnout, compassion fatigue, and moral injury during pandemics.^13^ It will be imperative for workplace colleagues to support each other and to perform frequent debriefs. Resources to support ED staff will vary by region, and they should be made easily accessible to all. Additionally, resources can be accessed through various licensing authorities and should be strongly encouraged.

## CONCLUSION

The workup and care of patients with COVID-19 already is, and will increasingly become, the role of the EP as the global pandemic evolves. EPs can also expect to care for patients who will be near the end of their lives due to this illness, and some of these patients will either not want or not benefit from escalating levels of care. This document and the associated online appendix provide a framework for end-of-life care that focuses on symptom management as well as minimizing risks of transmission to health care providers.

## Supplementary material

For supplementary material accompanying this paper visit http://dx.doi.org/10.1017/cem.2020.352. click here to view supplementary material
