# Supplementary material for: End-of-life care in the emergency department for the patient imminently dying of a highly transmissible acute respiratory infection (such as COVID-19)
Source: CJEM. 2020 Mar 26:1–4. doi: 10.1017/cem.2020.352 (PMC7138612; doi:10.1017/cem.2020.352)
Supplement: Supplementary file 1 [file S1481803520003528sup001.pptx]

## Slide 1
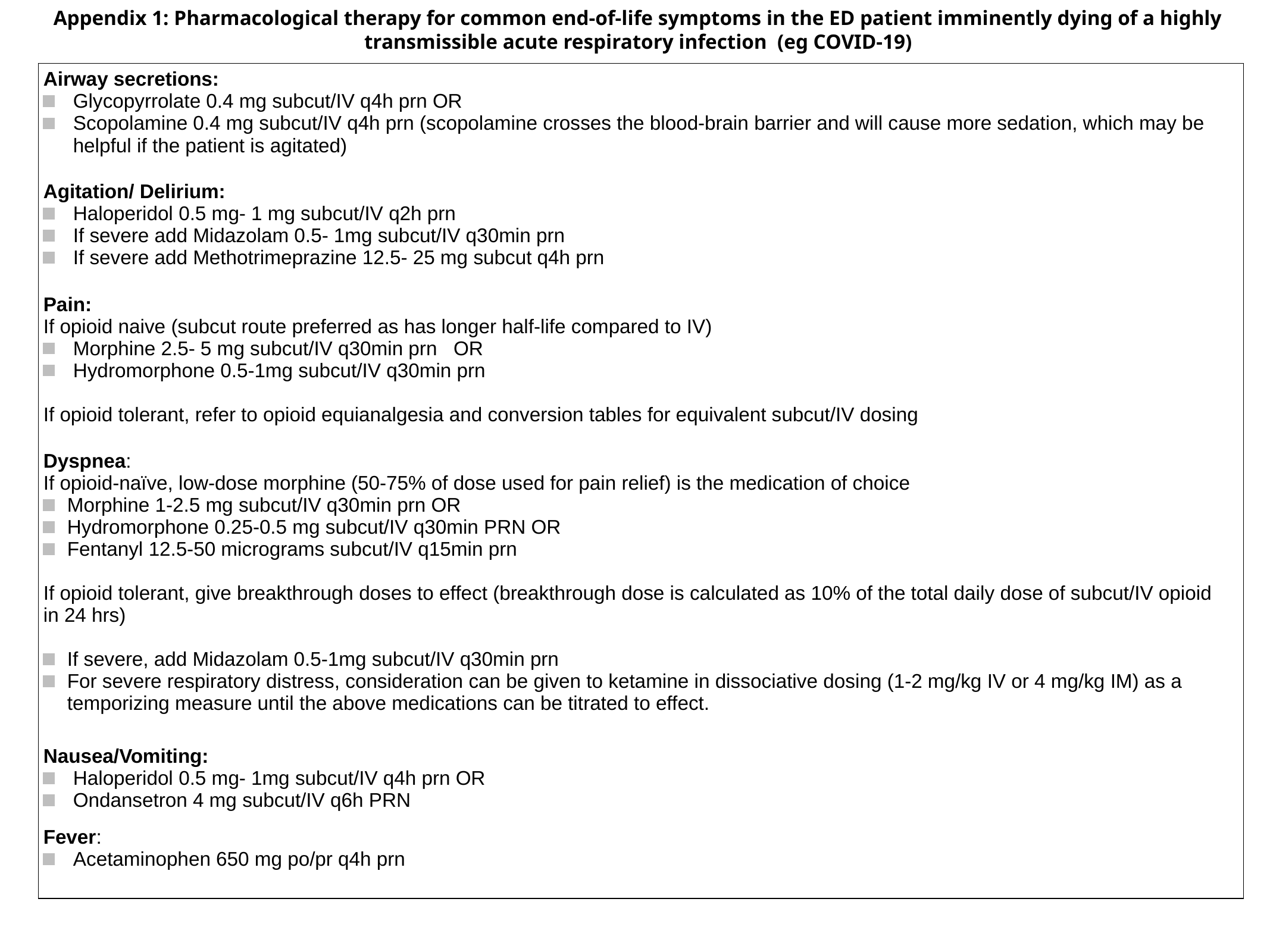

Appendix 1: Pharmacological therapy for common end-of-life symptoms in the ED patient imminently dying of a highly transmissible acute respiratory infection (eg COVID-19)
| Airway secretions: Glycopyrrolate 0.4 mg subcut/IV q4h prn OR Scopolamine 0.4 mg subcut/IV q4h prn (scopolamine crosses the blood-brain barrier and will cause more sedation, which may be helpful if the patient is agitated) |
| --- |
| Agitation/ Delirium: Haloperidol 0.5 mg- 1 mg subcut/IV q2h prn If severe add Midazolam 0.5- 1mg subcut/IV q30min prn If severe add Methotrimeprazine 12.5- 25 mg subcut q4h prn |
| Pain: If opioid naive (subcut route preferred as has longer half-life compared to IV) Morphine 2.5- 5 mg subcut/IV q30min prn OR Hydromorphone 0.5-1mg subcut/IV q30min prn If opioid tolerant, refer to opioid equianalgesia and conversion tables for equivalent subcut/IV dosing |
| Dyspnea: If opioid-naïve, low-dose morphine (50-75% of dose used for pain relief) is the medication of choice Morphine 1-2.5 mg subcut/IV q30min prn OR Hydromorphone 0.25-0.5 mg subcut/IV q30min PRN OR Fentanyl 12.5-50 micrograms subcut/IV q15min prn If opioid tolerant, give breakthrough doses to effect (breakthrough dose is calculated as 10% of the total daily dose of subcut/IV opioid in 24 hrs) If severe, add Midazolam 0.5-1mg subcut/IV q30min prn For severe respiratory distress, consideration can be given to ketamine in dissociative dosing (1-2 mg/kg IV or 4 mg/kg IM) as a temporizing measure until the above medications can be titrated to effect. |
| Nausea/Vomiting: Haloperidol 0.5 mg- 1mg subcut/IV q4h prn OR Ondansetron 4 mg subcut/IV q6h PRN |
| Fever: Acetaminophen 650 mg po/pr q4h prn |
